# Do the Benefits of Transplant Tourism Amongst Nigerian Patients Outweigh the Risks? A Single-Center Experience

**Published:** 2017-08-01

**Authors:** C. O. Amira, B. T. Bello

**Affiliations:** 1 Nephrology Unit, Department of Medicine, College of Medicine, University of Lagos, Idi-Araba, Lagos, Nigeria; 2 Nephrology Unit, Department of Medicine, College of Medicine, University of Lagos, Idi-Araba, Lagos, Nigeria

**Keywords:** Kidney transplantation, Medical tourism, Risk, Treatment outcome, Intraoperative complications, Postoperative complications

## Abstract

**Background:**

Transplant tourism (TT) is the term used to describe travel outside one’s country of abode for the sole purpose of obtaining organ transplantation services.

**Objective:**

This study describes the characteristics and outcomes of kidney transplant tourists who were followed up in our institution.

**Methods:**

A retrospective study was conducted on patients who underwent kidney transplantation outside the country and were followed up in our institution from 2007 to 2015.

**Results:**

26 patients were followed up; 19 (73%) were males. The mean±SD age of patients was 40.5±10.3 years. The majority (n=20) of the transplantations were carried out in India. Living-unrelated transplants were most common (54%). Complications encountered were infections in 11 (42%) patients, new-onset diabetes after transplantation in 9 (35%), chronic allograft nephropathy in 8 (31%), biopsy-proven acute rejections in 3 (12%), and primary non-function in 2 (8%). 1-year graft survival was 81% and 1-year patient survival was 85%.

**Conclusion:**

Kidney transplant tourism is still common among Nigerian patients with end-stage renal disease. Short-term graft and patient survival rates were poorer than values recommended for living kidney transplants. We therefore advise that TT should be discouraged in Nigeria, given the availability of transplantation services in the country, and also in line with international efforts to curb the practice.

## INTRODUCTION

Medical tourism has been defined as the practice whereby patients travel across national borders or overseas to another country for the sole purpose of medical treatment [1, 2]. Often times, people travel outside their country of residence for care because the treatment is not available within their home country; because of the perception that the quality of care at the destination country is superior to that at home; or because the care will be delivered in a timelier fashion compared to that in their home country [1-3]. While there are advantages like affordable costs, quality health care, and a chance to recuperate and have a vacation at the same time, there are also risks associated with medical tourism [3]. Among these are issues regarding safety of blood supply, quality of medications, complications arising from treatments, negligence, malpractice, and other ethical issues [2, 3].

Transplant tourism (TT) is the term used to describe travel outside one’s country of abode for the sole purpose of accessing organ transplantation services [4]. TT, unlike general medical tourism, is particularly plagued with potential clinical, legal, and ethical problems. In many cases, the source of donor organ is unknown; in some situations, the organs may be infected with blood-borne viruses, such as the human immunodeficiency virus (HIV), and the hepatitis B and C viruses, and still in other cases, the organs are clearly from commercial organ donors. Added to these, is the uncertainty about the quality of care, if any, that is available to the donors and in particular, recipient outcomes [4].

The first kidney transplant in Nigeria was carried out in March 2000 at a private facility in Lagos [5]. Since then the number of centers offering kidney transplantation in the country has risen to about 14 (Transplant Association of Nigeria Conference 2015). Despite the increasing availability of kidney transplantation services in Nigeria however, a good number of Nigerian patients with end-stage renal disease (ESRD) still travel abroad to be transplanted. This trend is further compounded by the fact that some Asian hospitals aggressively market kidney transplantation through “middlemen,” sometimes with the promise of providing donor kidneys for a fee. Anecdotal reports from several nephrologists in Nigeria suggest that patients who undergo kidney transplantation abroad often return with a myriad of health challenges and many do not follow up with their “home” nephrologists till they experience major complications. Empirical evidence regarding the health and safety risks facing Nigeria TT is however limited. 

The objective of this study was to determine the complications and outcomes of kidney transplant tourists managed in our institution.

## PATIENTS AND METHODS

This was a retrospective descriptive study of all kidney transplant recipients (KTRs) who underwent transplantation outside Nigeria and were subsequently followed up at the Nephrology Unit of a single teaching hospital in Lagos, Nigeria, over the last nine years, January 2007 to September 2015. Patients who were transplanted at any center within Nigeria were excluded. Information retrieved included patients’ demographics, original kidney disease, transplantation date and country of transplantation, the prescribed immunosuppressive regimen, serum creatinine at presentation, at one-year post-transplantation and the last follow-up visit, acute rejection episodes, and medical and surgical complications. 

Transplant outcomes were assessed as the graft and patient survival rates at one year and five years, as well as graft function at one year. Graft function at one year was considered “good” if serum creatinine was <133 µmol/L (1.5 mg/dL). 

Statistical Analysis

Data retrieved were analyzed using Epi Info^TM^ statistical software (CDC, Atlanta, USA). Quantitative data were presented as mean±SD, range, and median. Qualitative data were presented as percentages.

## RESULTS

**Figure 1:**
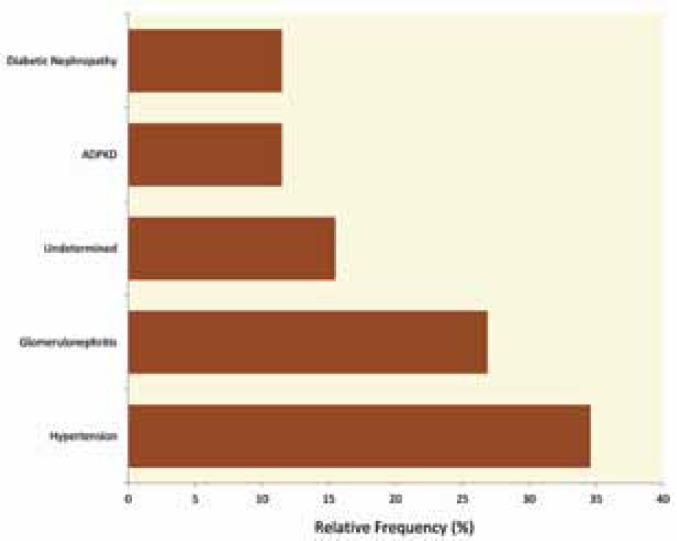
Primary renal disease leading to end-stage renal failure

Twenty six patients were followed up during the study period. Of these, 19 (73%) were males and 7 (27%) were females. The mean±SD age of the study participants was 40.5±10.3 (range: 18–65) years. Hypertension was the most common cause of ESRD (35%), followed by glomerulonephritis (27%) (Fig. 1). Two patients tested positive for antibodies to HIV-1 and were receiving antiretroviral therapy prior to transplantation. Two other patients tested positive for hepatitis-B surface antigen (HBsAg); neither receiving therapy for hepatitis B prior to transplantation. 

Twenty (77%) patients were transplanted in India, 4 (15%) in Pakistan and 2 (8%) were operated in Egypt. All 26 patients received living-donor kidneys; 14 (54%) received kidneys from unrelated donors while 12 (46%) received kidneys from related donors including two who received kidneys from emotionally related donors (spouses). Of the 14 patients who received kidneys from unrelated donors, 8 (31%) went with their donors from Nigeria while the remaining 6 (23%) donors were sourced from the country of transplantation, namely four from Pakistan and two from India. Eighteen (69%) patients travelled for transplantation without being referred by their doctors. Of the 26 kidney transplants, 17 (65%) were paid for by relatives of the recipient, 8 (31%) by the recipients themselves, and 1 (4%) by a religious organization. Table 1 shows the summary of clinical characteristics of the patients. 

**Table 1:** Clinical characteristics and outcomes of the patients. Figures are n (%) unless stated otherwise.

Variable	Statistics
Mean±SD age (yrs)	40.5±10.3
Male sex	19 (73%)
Type of transplant	
Living related	12 (46%)
Genetically related	10 (39%)
Brother	6
Mother	3
Son	1
Emotionally related	2 (8%)
Husband	1
Wife	1
Living unrelated	14 (54%)
Source of referral for transplantation	
No referral	18 (69%)
Doctor	8 (31%)
Median serum creatinine at 1^st^ clinic visit (µmol/L)	131.8
Median serum creatinine at last follow up visit (µmol/L)	456
1-year graft survival rate	21 (80.8)
1-year patient survival rate	22(84.6)

The median duration from time of transplantation to the initial follow-up visit was 8 weeks (range: 3 days to 28 weeks). The median duration from time of transplantation to this review was 3.8 (range: 1–8) years. Information on the induction immunosuppression employed in most of the patients was not available. However, at the time of initial post-transplantation clinic visit, 13 (50%) patients were on tacrolimus, mycophenolate mofetil (MMF), and prednisolone; 10 (39%) were on cyclosporine, MMF, and prednisolone; and 3 (12%) were on cyclosporine, azathioprine, and prednisolone. Three patients were later switched to a sirolimus-based protocol on account of chronic allograft nephropathy (n=2) and new-onset diabetes after transplantation (NODAT) (n=1). The median serum creatinine at the initial follow-up visit was 131.8 (range: 77.8–1228.8) µmol/L. The median serum creatinine at the last follow-up visit was 149 (range: 86–2519) µmol/L.

Complications

Complications experienced by the patients are summarized in Table 2. Infections were the most common complications occurring in 11 (42%) patients. These comprised sepsis (n=5) including one complicated by brain abscess, urinary tract infection (n=1), community-acquired pneumonia (n=1), wound infection (n=1), pulmonary tuberculosis (n=1), and cytomegalovirus (CMV) infection (n=1). One patient contracted hepatitis C infection. This patient was known to be seronegative prior to transplantation, and he subsequently developed progressive liver disease.

**Table 2:** Post-transplantation complications in the study population

Complications	n (%)
Infections	11 (42)
New-onset diabetes after transplantation (NODAT)	9 (35)
Chronic allograft nephropathy	8 (31)
Biopsy-proven acute rejection	3 (12)
Primary non-functioning allograft	2 (8)
Renal artery stenosis	1 (4)
Lymphocele	1 (4)

Three patients had biopsy-proven acute rejection; two of whom had HIV infection, both responding to treatment with steroids, but in one, graft function remained modestly impaired (serum creatinine 142 µmol/L); the other died later with a functioning graft 8 months post-transplantation from severe hyperglycemia and possible pulmonary embolism. Eight patients had chronic allograft nephropathy (CAN) of whom one experienced severe calcineurin inhibitor (CNI) toxicity. He did not present to a local nephrologist on initial return to the country after transplantation and therefore had no dose adjustments of his CNI. He presented seven months after transplantation when he developed symptoms of kidney failure. One patient developed a large lymphocoel post-transplantation. He returned to Nigeria within 72 hours of transplantation with all surgical drains and catheters in situ. The lymphocoel resolved following repeated ultrasound-guided percutaneous drainage and injection of a sclerosant.

Transplant Outcomes

Twenty two patients were alive after one year, giving a one-year patient survival of 85%. Causes of death were sepsis (n=2), and diabetic ketoacidosis complicated by suspected venous thromboembolism (n=1). Cause of death could not be ascertained in the fourth patient since he died at home but it is presumed to be related to complications of uremia. Five graft losses occurred within the first year, giving a one-year graft survival of 81%. Causes of graft loss were patient death (n=3) and primary non-functioning graft (n=2). Among the patients with a functioning graft one year post-transplantation, 13 (62%) had good graft function (defined as serum creatinine <133 µmol/L), with a median serum creatinine of 107 (range: 76–132 µmol/L). The remaining patients had serum creatinine level between 133 and 198 (median: 145) µmol/L. 

Duration since transplantation was five years or more in 12 patients. Amongst these, six were known to be dead; one had been lost to follow-up while five were alive giving a five-year patient survival of 42%. Of the five patients who were alive, four had functional grafts giving a five-year graft survival of 33%. Table 3 shows a comparison of transplant outcomes between the TT studied and the only nationwide data of locally transplanted patients in Nigeria. Both one-year and five-year graft and patient outcomes tended to be worse in the TT population. Among those who were not followed up to five years post-transplantation (n=14), 11 were alive and three dead, eight had functional grafts, while six grafts were lost.

**Table 3:** Comparison of transplant outcomes of transplant tourists with nationwide data of patents transplanted locally.

Transplant Outcomes	Transplant Tourist Population, (n=26)	Patients Transplanted within Nigeria, (n=143)
Acute rejection episodes	11.5%	15%–30%
1-year graft survival	80.8%	83.2%
1-year patient survival	84.6%	90.2%
5-year graft survival	33.3%	58.7%
5-year patient survival	41.7%	73.4%

Table 4 shows comparison of complications and outcomes of recipients of living-related transplant (LRT) and living-unrelated transplant (LURT). Tourist who had LRT had lower 1-year graft and patient survival. Only 33% of recipients of LRT had good function at one year. However, complications like infections, new-onset diabetes after transplantation (NODAT), and CAN were slightly higher among recipients of LURT. None of those who received LURT had biopsy confirmed acute rejection.

**Table 4:** Comparison of outcomes of LRT and LURT

Variable	LRT, (n=12)	LURT, (n=14)
Median age of recipients (yrs)	37.0	41.0
Source of referral for transplantation		
Self	7 (58%)	11 (79%)
Doctor	5 (42%)	3 (21%)
Median serum creatinine at 1^st^ visit (µmol/l)	137.0	114.9
Proportion with good graft function at 1 year	4 (33%)	9 (64%)
1-year graft survival	8 (67%)	13 (93%)
1-year patient survival	8 (67%)	14 (100%)
Complications		
Infections	5 (42%)	6 (43%)
NODAT	3 (25%)	6 (43%
Chronic allograft nephropathy	3 (25%)	5 (36%)
Biopsy proven acute rejection	3 (33%)	0 (0%)
Primary non-function	1 (8%)	1 (7%)
Lymphocele	0 (0%)	1 (7%)
Renal artery stenosis	0 (0%)	1 (7%)

LRT: Living-related transplants; LURT: Living-unrelated transplants; NODAT: New-onset diabetes after transplantation

At the end of the study period, a total of 13 (50%) graft losses had occurred; eight due to chronic allograft nephropathy (CAN), two due to primary non-functioning allograft, and three patients who died with functioning grafts. All patients with co-morbid hepatitis infections lost their grafts within 2–3 years of transplantation; two died from complications of progressive liver disease and one is back on hemodialysis. Sixteen (62%) patients were known to be alive, nine (35%) were dead, and one patient had been lost to follow-up. Of the 16 survivors, four were back on maintenance hemodialysis. 

## DISCUSSION

Kidney transplant is well known as the best form of treatment for ESRD, as it results in improvements in both the quality and quantity of life of the recipient [6]. In developed countries, donor kidneys are sourced mainly through deceased donor programs supplemented with living donation [7]. Conversely, in most developing countries, living donors continue to be the major source of transplanted kidneys due to the absence of deceased donor programs [4]. The severe shortage of donor kidneys in countries without deceased donor programs has paved the way for commercial, living unrelated kidney donation (LUKD) under the guise of TT [4, 8]. Commercial transplantation is estimated to account for 5%–10% of kidney transplants performed annually throughout the world [8]. There are concerns regarding the safety and outcomes of renal transplant tourists with several studies reporting worse outcomes among this group of kidney transplant recipients [4, 9-14]. Prior to March 2000, when the first kidney transplantation was carried out in Nigeria, all Nigerians who received transplanted kidneys had the procedure done outside the country because the facility was not available in Nigeria. Since then, an estimated 216 kidney transplantations have been carried out across 14 centers in the country (Transplant Association of Nigeria Conference 2015). Despite this, a good number of patients continue to engage in TT with its attendant health and safety risks and the potential problem of commercialization of kidney donation. 

There have been conflicting reports on the outcomes of TT worldwide. A recent systematic review of 27 publications from several countries revealed that although some studies reported good outcomes of LURT, the majority reported poor outcomes [15]. One of our notable findings was that the majority (54%) of the donor kidneys transplanted into the tourist population in this study were sourced from living unrelated donors. This contrasts starkly with the finding amongst locally transplanted patients in Nigeria where 82.5% of donor kidneys were sourced from genetically related donors [5]. Secondly, about 70% of the tourists travelled for transplantation without being referred by their managing nephrologist. This brings to the fore one possible reason for the decision to travel abroad for transplantation when such facilities are available in the home country. Although explanations such as the perception that facilities and expertise abroad are superior and are delivered in a more timely fashion, and at a more affordable cost (especially the cost of immunosuppressive medications) have often been advanced as a reason for the growth in TT; it may well be that transplant tourists are aware that many of the centers they travel to turn a blind eye to the issue of commercial kidney donation. 

Infections were the most common complications among our transplant tourists, occurring in 42% of the patients. This is consistent with most reports in the literature [9-12, 16]. Gills, *et* a*l*, reported infections among 52% of their transplant tourists with nine requiring hospitalization [9]. The type and nature of the infectious complications reported in previous studies vary. In our study population, bacterial infections were the most frequently seen infections. CMV infection was less common in our tourists than that reported in other studies, possibly because majority of the patients received CMV prophylaxis. Of note is the problem of viral hepatitis amongst our patients. Two patients were positive for HBsAg prior to transplantation and had been counselled about the need for treatment of hepatitis B before transplantation. Surprisingly both patients were transplanted first and one was not even commenced on therapy for hepatitis B post-transplantation. A third patient seroconverted for hepatitis C following transplantation. All three patients lost their grafts within 2–3years of transplantation. Surgical complications were a lot less common among our patients than has been reported in previous studies [9, 10, 16]. One possible reason for this may be because majority of our tourists stayed back in the country of transplantation for several weeks (median of 8 weeks) before returning home. Such complications had likely been dealt with prior to presenting to us and these were not often reported in the discharge summaries where available. This fact is further emphasized by the case of the patient who developed a large lymphocoel post-transplantation. He returned to Nigeria three days after being transplanted in Egypt with all drains and catheters in situ. Had he stayed longer in Egypt post-transplantation, the complication would most likely have been noticed before he presented to us.

Short-term graft and patient survival rates amongst our transplant tourist population were poorer than those published for patients transplanted locally within Nigeria [5]. One-year actual graft survival was 81% compared with 83.2% for patients transplanted locally, while one-year patient survival was 85% compared with 90.2% for patients transplanted locally. One-year graft survival in the tourist population was also lower than the World Health Organization (WHO) and The Transplantation Society estimates for both Africa (92%–95%) and South-East Asia (82%–87.5%) [17]. This finding of poorer short-term transplant outcomes for transplant tourists was similar to that reported by Kapour, *et* *al*, from Canada [10], and Krishnan, *et* *al*, from the UK [11]. It however contrasted with the findings by Gills, *et* *al* [9], who reported similar short-term outcomes between transplant tourists and patients transplanted in the Los Angeles, California and Bappa, *et al* [16], who reported good 1-year patient survival in the Kingdom of Saudi Arabia. 

Of particular concern was the finding that short-term outcomes (patient and graft survival rates) were worse among recipients of LRT compared with LURT. This raises questions about practices at the centers where these patients were transplanted. Majority of the patients travelled for transplantation without being referred by their doctors and possibly these patients were not adequately evaluated and prepared for surgery. We suspect that probably because the donors were related to the recipient, adequate care was not taken, especially in terms of induction therapy. Although all patients were on standard, triple immunosuppressive medication therapy consisting of a CNI, an anti-proliferative agents, and a corticosteroid at the time of initial follow-up visit, we had very little information on the induction therapy employed in the vast majority of the patients. One of the patients who had primary non-functioning graft received a kidney from a sibling with whom he shared a 4/6 human leukocyte antigen (HLA) match. Other complications such as infection rates and NODAT were however slightly more frequent among recipients of LURT.

Five-year graft and patient outcomes were also worse amongst the transplant tourist population whose duration since transplantation was longer than five years compared with patients transplanted locally in Nigeria (33% *vs*. 58.7%) and (42% *vs*. 73.4%), respectively [5]. We however, noted lower rates of acute rejection episodes (ARE) amongst the transplant tourist population compared with patients transplanted locally in Nigeria [5]. This finding differed significantly from that of Gills, *et* *al*, who reported ARE to occur almost twice as commonly in their transplant tourist population as in those transplanted locally at their center [9]. In our series, we identified only acute rejection episodes that were confirmed following graft biopsy. We suspect that this may be responsible for the apparently lower rates of ARE in the tourist population when compared to those patients transplanted locally.

Our study has some limitations including the small number of patients studied and lack of an ideal comparison group. The ideal comparison group would have been matched patients who received transplants locally at our center. However, our transplant program is still at its infancy with only four kidney transplants so far carried out and a one-year graft and patient survival of 100%. While conceding that the data on patients transplanted locally may be skewed by the fact that some centers have carried out only a few transplants with variable outcomes and that the two groups are not well matched, the consistent finding of poorer graft and patient outcomes, both at one and five years, is a signal that raises concern about the risk/benefit ratio of TT among Nigerian patients with ESRD. There is therefore a need for governments at all levels in Nigeria to provide support for renal care in general, and kidney transplantation in particular, in so as to enhance local transplant programs and reduce TT amongst ESRD patients. Enabling legislation needs to be passed to empower transplant units to commence deceased donor kidney transplant programs as a means of addressing the shortage of donor kidneys for transplantation. There is a need to increase the public awareness about the risks associated with TT, as well as encouraging altruistic kidney donation. The transplant centers may also explore the kidney transplantation model for developing countries as proposed by Ridzi, *et al* [18].

In conclusion, kidney TT is still common place among Nigerian patients with ESRD. Both short-term and long-term graft and patient outcomes appear to be poorer among transplant tourists (paradoxically more among recipients of LRT) than among patients transplanted locally. Transplant tourists and their donors may also be prone to exploitation. In tandem with international efforts to curb its practice, we therefore advise that TT should be discouraged in Nigeria given that transplant services are now readily available within the country with recipient outcomes that are, at the least, not inferior to those of patients travelling abroad for transplantation.
